# Contrast media-assisted in-treatment cone beam CT during single-isocentre volumetric-modulated arc therapy for multiple brain metastases: a case study

**DOI:** 10.1259/bjrcr.20160088

**Published:** 2016-09-07

**Authors:** Akihiro K Nomoto, Wataru Takahashi, Hideomi Yamashita, Akihiro Haga, Kiyoshi Yoda, Keiichi Nakagawa

**Affiliations:** ^1^Department of Radiology, University of Tokyo Hospital, Tokyo, Japan; ^2^Research Physics, Elekta KK, Tokyo, Japan

## Abstract

A direct visualization technique for verifying intrafractional localization accuracy of multiple brain metastases during single-isocentre volumetric-modulated arc therapy has been proposed using contrast media-assisted in-treatment cone beam CT (CBCT). Contrast-enhanced planning CT images were acquired immediately after intravenous bolus administration of iodized contrast media at a dose of 2 ml kg^–1^. Out of 41 nodules detected on the images, 8 lesions were contoured as high-risk gross tumour volumes (GTVs). Prior to each treatment, CBCT imaging was performed to match the skull structures with the planning CT images. Immediately after another intravenous bolus injection of the iodized contrast media at the same dose as administered for the planning CT imaging, contrast-enhanced CBCT images were acquired during volumetric-modulated arc therapy delivery, thereby providing direct verification of time-averaged tumour position during treatment. The planning target volume contours were overlaid with the in-treatment CBCT images, thereby allowing us to directly visualize the localization accuracy of each GTV when the beam delivery was completed. It was visually confirmed that each GTV was accurately localized inside each planning target volume during beam delivery.

## Introduction

Contrast-enhanced CT scan has a long history of being used for visualizing brain lesions.^1^ It was later applied to on-board cone-beam CT (CBCT) equipped with a linear accelerator for localizing brain tumours prior to stereotactic treatment in 2008.^2,3^ The contrast-enhanced CBCT imaging prior to treatment facilitated visual confirmation of the localization accuracy because brain tumours were invisible without administration of the contrast media. However, the localization accuracy may be decreased during treatment. Meanwhile, intrafractional CBCT was also clinically demonstrated to verify tumour positions during volumetric-modulated arc therapy (VMAT) for tumours of the prostate^4^ and lungs.^5,6^ Naturally, the next step was to combine these two techniques. In particular, when the single-isocentre VMAT is employed for the multiple brain tumours,^7^ localization accuracy of the tumours located distant from the isocentre may be of greater concern owing to rotational positioning errors.^8,9^

The purpose of this study was to propose a direct procedure for verifying the accuracy of brain tumour localization by way of intrafractional contrast-enhanced CBCT. To the authors’ knowledge, this has not been reported previously.

## Materials and methods

### Patient characteristics

A 59-year-old female patient had a 17-year history of breast cancer with diagnosis of multiple brain metastases. After receiving breast conserving therapy as primary treatment, the patient was diagnosed with local recurrence and multiple lung metastases 12 years later. Immunohistochemical tests were positive for oestrogen receptors, but negative for progesterone receptors and Her2-neu. Progressive disease and low adherence resulted in discontinuation of salvage treatments. After the progression of multiple brain metastases, the patient was referred to our hospital for radiation therapy. Even with multiple metastases to the skin, lung, adrenal grand and liver, her Karnofsky performance status was 90%.

### Gross tumour volume delineation

An intravenous bolus injection of iodized contrast media (Iopamiron 300, Schering, Berlin, Germany) was manually administered at a dose of 2 ml kg^–1^ and an injection time of 90 s. 30 s after injection of the contrast media, contrast-enhanced planning CT images were acquired by a large bore CT scanner, Aquilion LB (Toshiba Medical Systems, Tokyo, Japan), with an exposure of 175 mAs. Out of 41 nodules detected on the images, eight lesions were contoured as high-risk gross tumour volumes (GTVs) because seven of them had a volume larger than 1 ml and one nodule having a volume of 0.7 ml was located in proximity to the brainstem.

### Treatment planning

A coplanar two-arc VMAT plan was created by Monaco treatment planning system, version 5.0 (Elekta, Stockholm, Sweden), with a prescribed dose of 30 Gy in 10 fractions to the whole brain and a total dose of 45 Gy in 10 fractions to the high-risk GTVs by simultaneous integrated boost. In principle, the margin for each tumour needs to take into account both translational and rotational localization errors. In other words, the translational error in each axis may require a spatially invariant margin, while the rotational error around each axis, especially when a six-degrees-of-freedom couch is not available, may need additional margins proportional to the distance between each tumour and the single isocentre. Our couch had only three degrees of freedom; therefore, for simplicity, two different planning target volume (PTV) margins for the eight high-risk GTVs were employed depending on the distance between each target and the isocentre: 2 mm for the targets located within 5 cm from the isocentre and 3 mm for the other targets located within 10 cm from the isocentre.

### Skull registration prior to treatment

A thermoplastic mask combined with a mouthpiece, Precise Bite (CIVCO Medical Solutions, Kalona, IA), was employed to immobilize the patient's head. Subsequently, CBCT images were acquired by kilovoltage X-ray volume imaging, version 5.0 (Elekta), to match the skull structure with the planning CT images. A three-degrees-of-freedom couch, PreciseTable (Elekta), was adjusted according to the measured registration errors. If the rotation angle error was larger than 1°, a technologist adjusted the orientation of the patient's head accordingly and the registration was performed again.

### Treatment delivery

Prior to the treatment, informed consent had been obtained from the patient. After the treatment plan passed the quality assurance test, the patient was treated by an Elekta research linear accelerator with an Agility multileaf collimator (Elekta).

### Intrafractional contrast-enhanced CBCT imaging

Immediately after another intravenous bolus injection of the iodized contrast media at the same dose as administered for the planning CT imaging, contrast-enhanced intrafractional CBCT images were acquired during the first arc of the two-arc VMAT delivery, with a typical imaging dose of 25 mGy. As the administration of contrast-enhanced media in all fractions could influence kidney function severely, we acquired the contrast-enhanced CBCT in only one fraction. The intrafractional CBCT imaging functionality was provided by the X-ray volume imaging system.

### Verification of intrafractional target positions

Immediately after the beam delivery, intrafractional target positions shown on the contrast-enhanced CBCT images and the overlaid PTV contours were visually compared to verify the accuracy of target localization.

## Results

**Figure 1.**
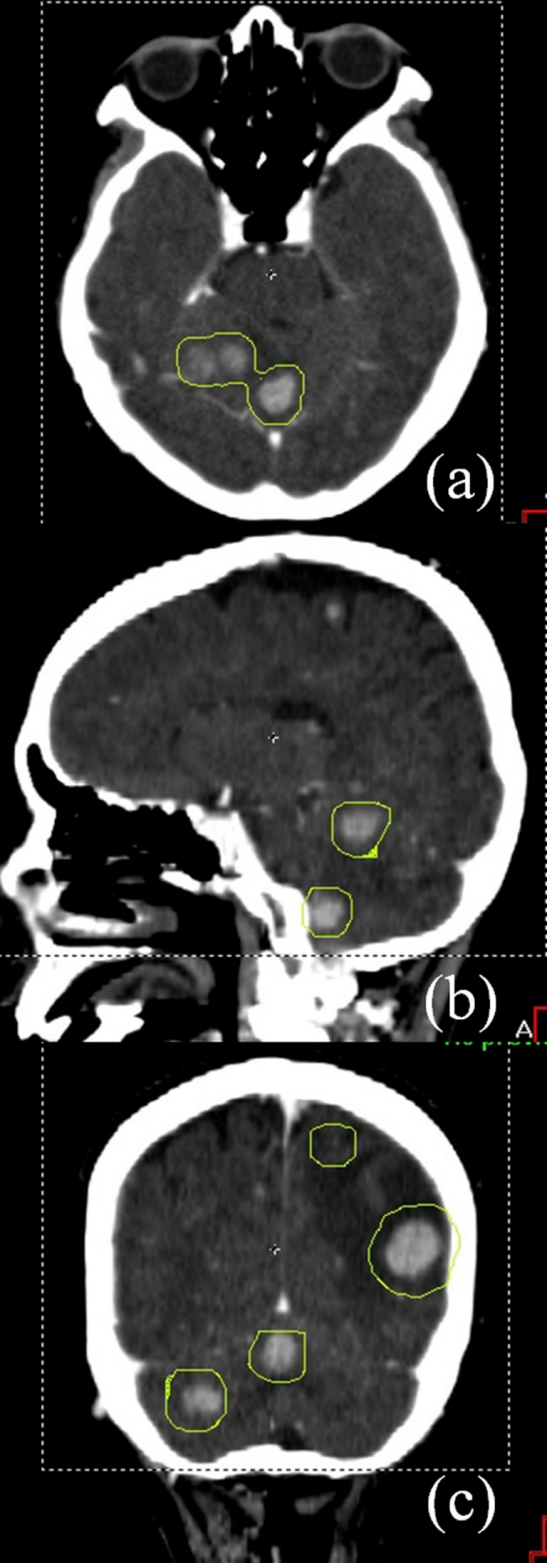
Contrast-enhanced planning CT images on (a) axial, (b) sagittal and (c) coronal planes, with eight lesions contoured as high-risk gross tumour volumes. Planning target volume margins were 2 mm for the targets located within 5 cm from the isocentre, and 3 mm for the other targets located within 10 cm from the isocentre. The resulting planning target volume contours are shown in light green colour.

**Figure 2.**
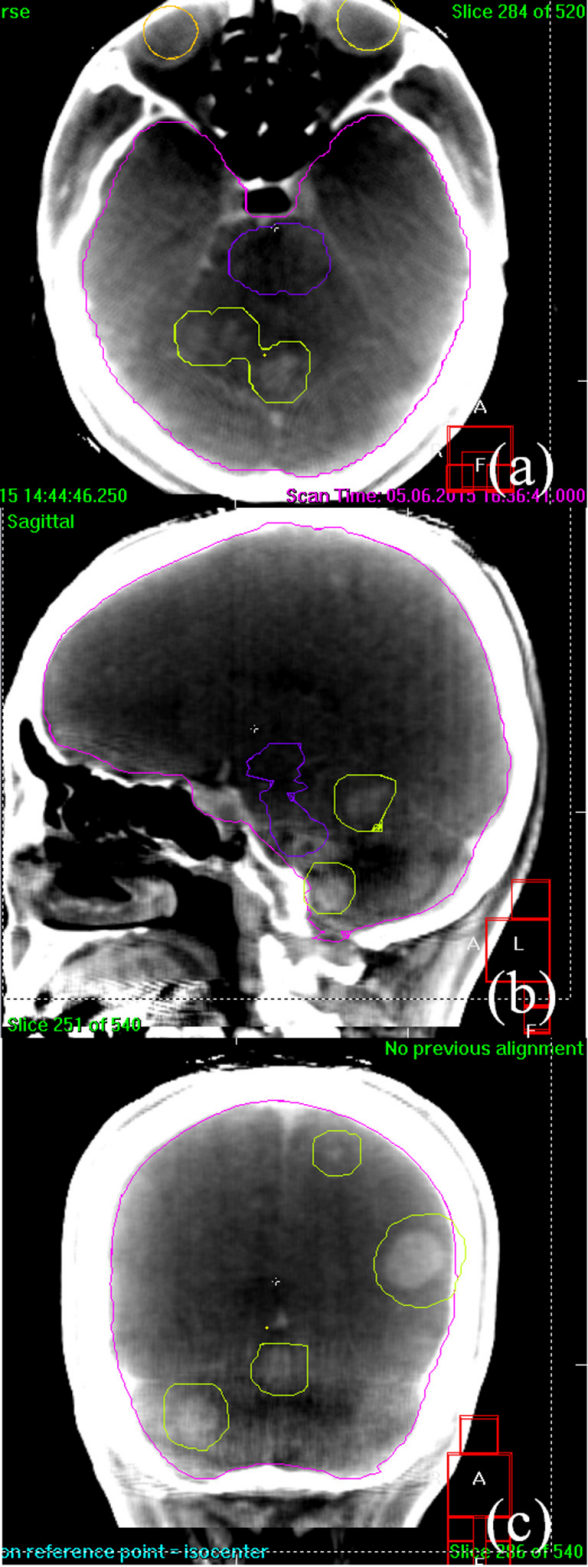
Contrast-enhanced in-treatment cone-beam CT images on (a) axial, (b) sagittal and (c) coronal planes during single-isocentre volumetric-modulated arc therapy acquired immediately after another intravenous bolus injection of iodized contrast media at the same dose as administered for the planning CT imaging. The planning target volume contours were also overlaid, thereby allowing us to directly verify the localization accuracy of each gross tumour volume.

**Figure 3.**
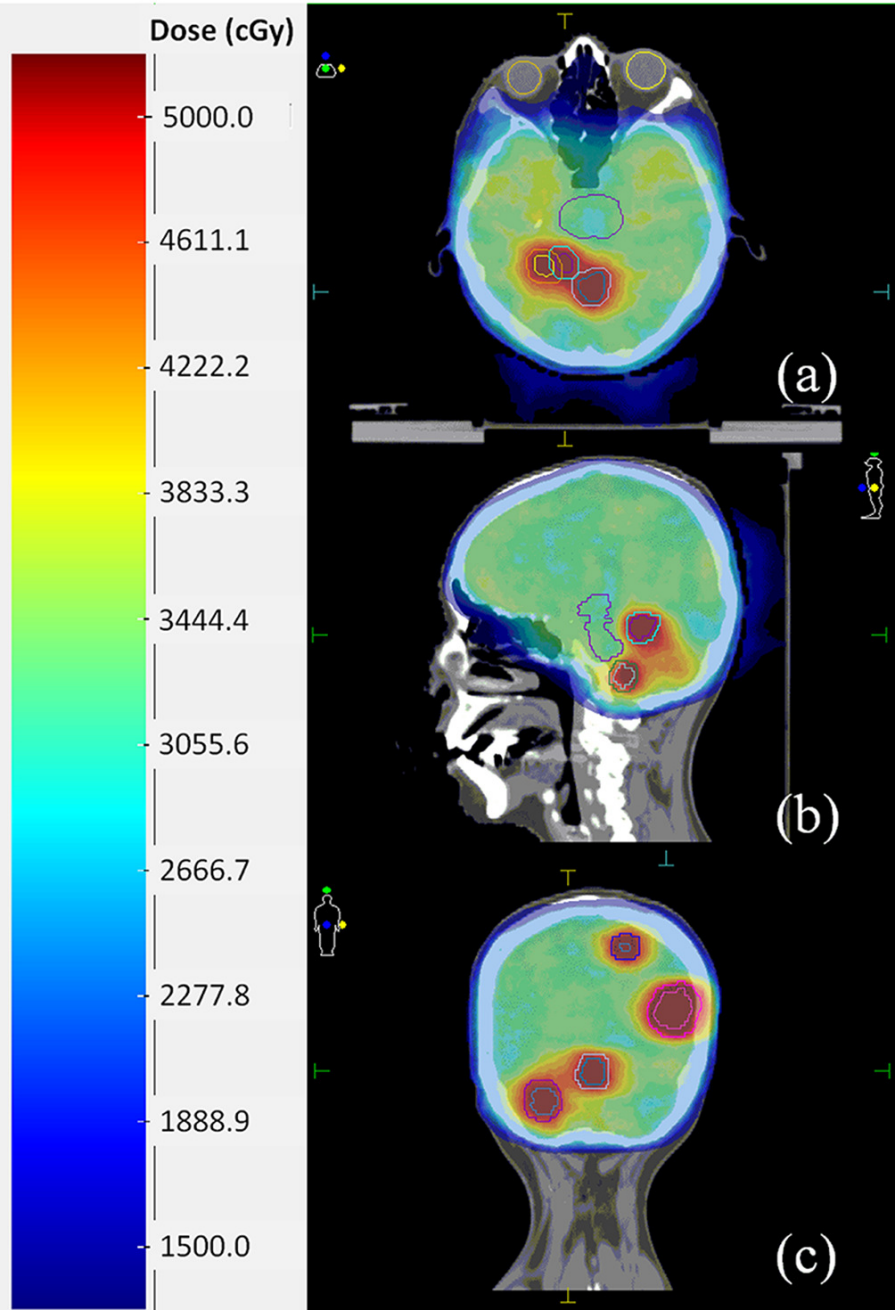
Calculated dose distributions resulting from a coplanar two-arc volumetric-modulated arc therapy plan with a prescribed dose of 30 Gy in 10 fractions to the whole brain and a total dose of 45 Gy in 10 fractions to the high-risk gross tumour volumes by simultaneous integrated boost.

Figure 1 shows the contrast media-assisted planning CT images in (a) axial, (b) sagittal, and (c) coronal planes. The PTV contours are also shown. Figure 2 depicts contrast media-assisted in-treatment CBCT images in (a) axial, (b) sagittal, and (c) coronal planes during the single-isocentre VMAT delivery. The PTV contours were also overlaid. The beam on time for the first arc was 2 min 19 s while that for the second arc was 3 min 15 s. Figure 3 shows calculated dose distributions resulting from the VMAT plan, with a prescribed dose of 30 Gy in 10 fractions to the whole brain and a total dose of 45 Gy in 10 fractions to the high-risk GTVs by simultaneous integrated boost.

## Discussion

It was reported that the rotational positioning accuracy may be highly arguable for single-shot stereotactic radiosurgery because a significantly large rotational positioning error may arise as a statistical event during treatment^8,9^ It is our intention to calculate in-treatment tumour localization accuracy for each metastasis by a direct three-dimensional visualization technique rather than a single estimation from a skull bone matching result. The initial experience shown here seems promising and a further study should be conducted, including investigation of the optimal contrast media dose and CBCT exposure conditions, with a relatively larger patient population.

## Conclusions

A direct visualization technique for verifying intrafractional localization accuracy of multiple brain metastases during single-isocentre VMAT has been proposed using contrast media-assisted in-treatment CBCT. The PTV contours were overlaid with the in-treatment CBCT images, thereby allowing us to directly visualize each GTV in reference to each PTV position when the beam delivery was completed. It was confirmed that each GTV was accurately localized inside each PTV during beam delivery. As a future perspective, the results may be adaptively utilized for the remaining fractional treatment planning, including the PTV margin adjustment.

## Consent

Written informed consent for the case to be published (including images, case history and data) was obtained from the patient(s).

## References

[1] Davis KR, New PF, Solis OJ, Roberson GH. A review of the findings on computed cranial tomography following intravenous contrast media. Rev Interam Radiol 1977; 2: 15–18. 866885

[2] Nakagawa K, Yamashita H, Igaki H, Terahara A, Shiraishi K, Yoda K. Contrast medium-assisted stereotactic image-guided radiotherapy using kilovoltage cone-beam computed tomography. Radiat Med 2008; 26: 570–2. 19030968 10.1007/s11604-008-0275-2

[3] Igaki H, Nakagawa K, Yamashita H, Terahara A, Haga A, Shiraishi K, et al Contrast media-assisted visualization of brain metastases by kilovoltage cone-beam CT. Acta Oncol 2009; 48: 314–7. 18759140 10.1080/02841860802310983

[4] Nakagawa K, Haga A, Shiraishi K, Yamashita H, Igaki H, Terahara A, et al First clinical cone-beam CT imaging during volumetric modulated arc therapy. Radiother Oncol 2009; 90: 422–3. 19062117 10.1016/j.radonc.2008.11.009

[5] Takahashi W, Yamashita H, Kida S, Masutani Y, Sakumi A, Ohtomo K, et al Verification of planning target volume settings in volumetric modulated arc therapy for stereotactic body radiation therapy by using in-treatment 4-dimensional cone beam computed tomography. Int J Radiat Oncol Biol Phys 2013; 86: 426–31. 23562767 10.1016/j.ijrobp.2013.02.019

[6] Nakagawa K, Haga A, Kida S, Masutani Y, Yamashita H, Takahashi W, et al 4D registration and 4D verification of lung tumor position for stereotactic volumetric modulated arc therapy using respiratory-correlated cone-beam CT. J Radiat Res 2013; 54: 152–6. 22843380 10.1093/jrr/rrs058 PMC3534265

[7] Mayo CS, Ding L, Addesa A, Kadish S, Fitzgerald TJ, Moser R. Initial experience with volumetric IMRT (RapidArc) for intracranial stereotactic radiosurgery. Int J Radiat Oncol Biol Phys 2010; 78: 1457–66. 20207494 10.1016/j.ijrobp.2009.10.005

[8] Roper J, Chanyavanich V, Betzel G, Switchenko J, Dhabaan A. Single-isocenter multiple-target stereotactic radiosurgery: risk of compromised coverage. Int J Radiat Oncol Biol Phys. 2015; 93: 540–6. 26460996 10.1016/j.ijrobp.2015.07.2262 PMC4610743

[9] Gevaert T, Verellen D, Engels B, Depuydt T, Heuninckx K, Tournel K, et al Clinical evaluation of a robotic 6-degree of freedom treatment couch for frameless radiosurgery. Int J Radiat Oncol Biol Phys 2012; 83: 467–74. 21945110 10.1016/j.ijrobp.2011.05.048

